# Exosome-Based Drug Delivery: Translation from Bench to Clinic

**DOI:** 10.3390/pharmaceutics15082042

**Published:** 2023-07-29

**Authors:** Hee Byung Koh, Hyo Jeong Kim, Shin-Wook Kang, Tae-Hyun Yoo

**Affiliations:** 1 Division of Nephrology, Department of Internal Medicine, International Saint Mary’s Hospital, College of Medicine, Catholic Kwandong University, Seo-gu, Incheon 22711, Republic of Korea; kjcvkjcv@gmail.com; 2 Division of Nephrology, Department of Internal Medicine, Gangnam Severance Hospital, College of Medicine, Yonsei University, Gangnam-gu, Seoul 06273, Republic of Korea; tojeong@yuhs.ac; 3 Department of Internal Medicine, Institute of Kidney Disease Research, College of Medicine, Yonsei University, Seodaemun-gu, Seoul 03722, Republic of Korea

**Keywords:** exosome, extracellular vesicle, drug delivery

## Abstract

Exosome-based drug delivery is emerging as a promising field with the potential to revolutionize therapeutic interventions. Exosomes, which are small extracellular vesicles released by various cell types, have attracted significant attention due to their unique properties and natural ability to transport bioactive molecules. These nano-sized vesicles, ranging in size from 30 to 150 nm, can effectively transport a variety of cargoes, including proteins, nucleic acids, and lipids. Compared to traditional drug delivery systems, exosomes exhibit unique biocompatibility, low immunogenicity, and reduced toxicity. In addition, exosomes can be designed and tailored to improve targeting efficiency, cargo loading capacity, and stability, paving the way for personalized medicine and precision therapy. However, despite the promising potential of exosome-based drug delivery, its clinical application remains challenging due to limitations in exosome isolation and purification, low loading efficiency of therapeutic cargoes, insufficient targeted delivery, and rapid elimination in circulation. This comprehensive review focuses on the transition of exosome-based drug delivery from the bench to clinic, highlighting key aspects, such as exosome structure and biogenesis, cargo loading methods, surface engineering techniques, and clinical applications. It also discusses challenges and prospects in this emerging field.

## 1. Introduction

Exosomes are extracellular vesicles (EVs) that are secreted by various cell types [1]. These vesicles typically have a diameter of 30–150 nm and contain a wide range of bioactive cargoes, including proteins, lipids, deoxyribonucleic acid (DNA), small interfering ribonucleic acid (siRNA), messenger RNA (mRNA), microRNA (miRNA), and various signaling molecules. These cargoes, packed within lipid bilayers, can be transferred to recipient cells, altering their behavior and playing a critical role in cell-to-cell communication. Based on these theoretical foundations, exosomes have gained increasing attention over the past decade as promising drug delivery platforms. The use of exosomes has several advantages over conventional drug delivery systems, such as polymeric nanoparticles, dendrimers, micelles, and liposomes [2]. For example, liposomes, the most widely studied representative drug delivery vehicles, are also composed of phospholipid bilayers. Conventional liposomes lack target specificity, rely on passive accumulation to deliver drugs to specific tissues, and are vulnerable to clearance by the mononuclear phagocyte system (MPS), resulting in poor efficacy. To overcome this limitations, various kinds of functionalized liposomes, such as cationic liposomes, long-circulating liposomes using polyethylene glycol (PEG), ligand-targeted liposomes, and temperature- and pH-sensitive liposomes, were investigated [3,4,5,6]. However, liposomes still face several challenges, including the requirement for sophisticated equipment and high production costs, short half-life, potential immunotoxicity, and issues related to the instability of phospholipid components or denaturation of encapsulated drugs during the manufacturing process [7,8,9]. However, exosomes occur naturally in the body and exhibit low immunogenicity and toxicity. They can bypass the endosomal pathway and avoid lysosomal degradation, enabling the direct delivery of cargo into the cytoplasm [10]. Additionally, exosomes can easily cross biological barriers, such as the blood–brain barrier, which is one of the major challenges of conventional drug delivery systems [11]. They are naturally derived from cells and possess inherent targeting capabilities that allow them to deliver therapeutic cargo to specific tissues or cells, thereby reducing systemic toxicity. Moreover, exosomes can be engineered to target specific cells using ligands, magnetic materials, and pH-responsive motifs, which can enhance drug delivery specificity and efficacy [12]. However, despite the potential of exosome-based drug delivery, its clinical application remains challenging owing to the limitations of exosome isolation and purification, low loading efficiency of therapeutic cargoes, insufficient targeted delivery, and elimination via reticuloendothelial systems [13,14,15]. This comprehensive review covers exosome-based drug delivery, the structure and biogenesis of exosomes, drug-loading approaches, surface engineering, and clinical applications of exosome-based drug delivery, as well as the challenges and prospects for this emerging field.

## 2. Structure and Biogenesis of Exosomes

### 2.1. Exosome Structure

**Figure 1:**
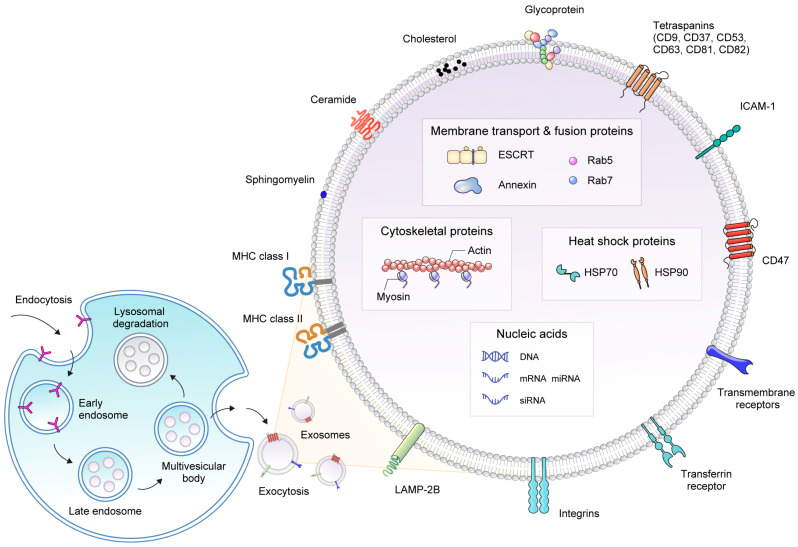
Structure and biogenesis of exosome.

Exosomes, a type of EV, exhibit unique characteristics compared with other EVs, such as microvesicles (MVs) and apoptotic bodies, that contribute to their functional diversity. One notable distinction lies in their size. Exosomes typically range from 30 to 150 nm in diameter, making them smaller than MVs (100–1000 nm), and have a narrower size range than apoptotic bodies (50–5000 nm). This specific size range enables the convenient isolation and characterization of exosomes using techniques such as ultracentrifugation, size-exclusion chromatography, and specialized nanoparticle tracking analysis [16]. Another key aspect of exosome structure is the lipid bilayer membrane (Figure 1). Similar to other EVs, exosomes are enclosed in a phospholipid bilayer resembling the plasma membrane of the parent cell. The lipid membrane provides stability and protection to the encapsulated cargo, shielding it from degradation by the extracellular environment [12]. The lipid composition of exosomes can vary depending on the parent cell type and physiological conditions and influences their biophysical properties and interactions with recipient cells. The protein composition also provides clues for distinguishing exosomes from other EVs. Exosomes are enriched with specific proteins, such as tetraspanins (CD9, CD63, and CD81), which are commonly used as exosome markers [17]. These tetraspanins play crucial roles in various cellular processes, including multivesicle biosynthesis, cell migration, cell-to-cell adhesion, and signaling. Additionally, certain integrin-associated transmembrane proteins, such as CD47, have been identified as key factors that enhance the stability of exosomes by restricting their clearance from mononuclear phagocyte system [18]. Heat shock proteins (HSP70 and HSP90), various receptors, and adhesion molecules are often found on the exosome surface, contributing to their targeting and uptake by recipient cells [19]. However, the expression of these proteins is not exclusive to exosomes because they can also be detected in other types of vesicles. Exosomes contain various biomolecules, including proteins, lipids, DNA, RNA, and small non-coding RNAs. These cargo molecules are selectively packaged and sorted into exosomes via various mechanisms [20]. This unique cargo composition enables exosomes to transport molecular information to recipient cells, resulting in various physiological effects, and perform the role of transporter for drug delivery.

### 2.2. Exosome Biogenesis

Exosome biogenesis begins with the endocytic pathway, whereby endocytic vesicles are formed by the invagination of the plasma membrane. These internalized vesicles are sorted into early endosomes and matured into late endosomes. Late endosomes can subsequently transform into multivesicular bodies (MVBs) via inward budding of the endosomal membrane, resulting in the formation of intraluminal vesicles (ILVs) within MVBs [21]. Various proteins are incorporated into them. Ultimately, MVBs fuse with the plasma membrane of the cells, resulting in the release of exosomes [22]. ILV biogenesis is a complex process that involves several coordinated protein complexes. The endosomal sorting complex required for transport (ESCRT) is a key protein involved in ILV formation. The ESCRT machinery consists of four subunits, ESCRT-0, ESCRT-I, ESCRT-II, and ESCRT-III, which work together to sort ubiquitinated cargo into ILVs [23]. First, ESCRT-0 recognizes and binds to the ubiquitinated cargo on the endosomal membrane. ESCRT-I and ESCRT-II bind to the cargo and recruit ESCRT-III. Subsequently, the nucleated ESCRT-III assembly recruits the deubiquitination machinery and drives the inward budding of the endosomal membrane. Finally, the Vps4-Vta1 complex completes vesicle budding via scission. There are also ESCRT-independent pathways for cargo-sorting systems, the activity of which is governed by lipids, including ceramide and sphingomyelin [24]. High concentrations of sphingolipids induce the formation of ceramides via neutral sphingomyelinase (nSMase), which is involved in cargo sorting during exosome biogenesis [25]. The inhibition of SMase2 or specific knockdown of its expression decreases the secretion of miRNA-loaded exosomes, indicating the involvement of nSMase-dependent pathways rather than the endosomal sorting complex. This is supported by evidence from a study showing that cargo is sorted into ILVs when the intracellular ESCRT protein is depleted, even though the shape of the endocytic structures is dramatically altered, indicating a coordinated exosome biogenesis process involving both ESCRT-dependent and ESCRT-independent pathways [26]. The tetraspanin family, including CD81, CD9, and CD63, plays a significant role in protein cargo selection during exosome biogenesis. Indeed, the lack of CD81 reduces the presence of CD81-related proteins, such as Rac, in exosomes [27]. Additionally, the knockdown of CD9 expression using short hairpin RNA results in a twofold reduction in the release of endogenous CD10 with MVs [28]. Several other proteins are involved in the cargo sorting of exosomes, including the RNA-binding protein heteronuclear ribonucleotide, major bolt protein, Y-box protein 1, and Nedd4 family interacting protein 1.

## 3. Cellular Source of Exosome and Isolation Methods

### 3.1. Cellular Source of Exosome

Exosomes can be derived from a variety of cellular sources, and each source has unique properties that may impact clinical applications when used for drug delivery vehicles or therapeutics on their own. Therefore, one of the initial requirements for developing an efficient exosome-based drug delivery system is the selection of an optimal donor cell type. Human embryonic kidney (HEK) cells are commonly used in research for the production of exosomes due to their ability to yield high quantities of exosomes, ease of cultivation, and low toxicity and minimal immune response [29]. Exosomes-derived from HEK cells have little involvement in physiological and pathological processes, highlighting their suitability as drug delivery vehicles within the body [30]. In addition, although they exhibit membrane characteristics resembling epithelial membranes, they also display similar protein distribution in various tissues of the body, including platelet, lymph, liver, brain, lung, and muscle. While there is an inherent risk of off-target effects, the broad tissue distribution similarity of HEK cell-derived exosomes suggests their potential for universal applicability in targeting diverse tissues. Moreover, HEK cells exhibit high transfection efficiency, making it easy to load therapeutic small RNAs and modify exosomes through transfection [30]. Exosomes derived from immune cells inherit the characteristics of their parent cells, allowing them to actively participate in both innate and adaptive immune responses. This quality makes them a highly promising option for drug delivery in cancer immunotherapy research. For instance, dendritic cell-derived exosomes possess essential immune-stimulatory components, such as major histocompatibility (MHC) I and MHC II complexes, as well as abundant costimulatory molecules [31]. These exosomes can be loaded with neoantigens and ovalbumin, enabling their utilization in cancer immunotherapy as a cell-free alternative to address the limitations of dendritic cell-based immunotherapy [32]. Mesenchymal stem cells (MSCs) can also be utilized as donor cells for exosome production. MSC-derived exosomes possess inherent anti-fibrotic, anti-inflammatory, and angiogenesis-promoting properties in several tissues, such as myocyte, kidney, and liver [33,34,35]. This characteristic of MSC-derived exosomes enables them not only to serve as a platform for drug delivery, but also to exert their own therapeutic effects. However, MSCs have limited proliferation for large-scale production, and batch-to-batch variations can occur in exosome production [36]. To overcome these obstacles, c-myc transfected human embryonic stem cell-derived MSCs (hESC-MSCs) have been investigated. These modified cells have shown enhanced proliferation rates and reduced production time, while maintaining exosome quality. In a comparative study, c-myc transfected hESC-MSCs showed at least a tenfold increase in exosome production compared to other cell lines, such as the skeletal muscle cell line, HEK cells, small airway epithelial cell line, and human leukemia monocytic cell line [37]. Tumor cell-derived exosomes are commonly utilized as diagnostic biomarkers, rather than drug delivery vehicles, due to the potential presence of oncogenic signals derived from the parent tumor cells [38,39]. However, these exosomes contain immune-stimulatory molecules that can act as adjuvants for antigen presentation and immune response activation. Therefore, tumor cell-derived exosomes have the potential to enhance the anti-tumor response by engaging immune cells, like neutrophils, thereby amplifying the therapeutic effect when delivering anti-cancer drugs to target cancer cells, leveraging their distinctive tropism [40].

### 3.2. Isolation Methods for Exosome

Exosome isolation is a critical step in the field of exosome-based drug delivery. Obtaining pure exosome populations from biological fluids or cultured media can be challenging due to their small size and the presence of other EVs and contaminants. Several methods are commonly used for exosome isolation, each with its advantages and limitations. Sequential ultracentrifugation, one of the most widely used techniques, involves sequential centrifugation steps to pellet exosomes based on their density, making it ideal for large-scale samples and cost-effective [41]. Furthermore, it poses a low risk of contamination from additional isolation reagents. However, the method has drawbacks, such as being time-consuming, being labor-intensive, requiring specialized equipment, and potentially causing exosome damage [42]. The density gradient centrifugation method is employed to further separate exosomes based on their density in a dense medium [43]. This approach prevents exosomal damage, resulting in high purity; however, the yield of isolated exosomes may be minimal. In addition, the procedure is time-consuming, and the preliminary preparation can be labor-intensive [42]. Ultrafiltration is a method that employs filters with specific pore sizes to concentrate exosomes while eliminating larger particles [44]. This technique is relatively straightforward and does not necessitate any special equipment or reagents, making it cost-effective. Moreover, the process can be completed in a relatively short time, usually taking less than 4 h. It yields exosomes with high purity and provides a moderate overall yield. However, it is essential to consider that exosomes with tiny particle diameters might be lost during the ultrafiltration process due to potential clogging on the filtering membrane. Size-exclusion chromatography utilizes a porous gel polymer as the stationary phase and the starting biofluid as the moving phase to elute particles, with larger vesicles eluting first, followed by smaller vesicles [45]. This method offers high purity and quick preparation, preserving the native state of exosomes. However, it is essential to consider the high device cost and the possibility of potential lipoprotein contamination [46]. Immunoaffinity utilizes specific antibodies that interact with particular membrane proteins of exosome, enabling the separation of exosomes of specific origin with high purity [43]. However, it is limited by low processing volume and yields. Furthermore, the effectiveness depends on the specificity of the antibodies used [47]. Precipitation method captures exosome sized between 50 and 150 nm in polymer nets using low-speed centrifugation. The addition of polymeric components alters the solubility and partitioning properties of exosomes, resulting in precipitation [48]. This method offers benefits in terms of simplicity and high efficiency. However, it has disadvantages, such as the risk of protein aggregate and polymeric contaminations, as well as extended processing time [49].

## 4. Drug-Loading Approaches to Exosomes

Incorporating therapeutic cargo into exosomes presents a promising avenue for substantially improving therapeutic efficacy by preserving its integrity within the in vivo environment and enhancing its biodistribution. Exosomes possess a distinctive structural framework that allows the encapsulation of hydrophilic cargo within their core, whereas hydrophobic substances are encapsulated within the lipid bilayer [50]. Various approaches have been developed to successfully encapsulate drugs in exosomes, paving the way for the development of advanced therapeutic strategies.

**Figure 2:**
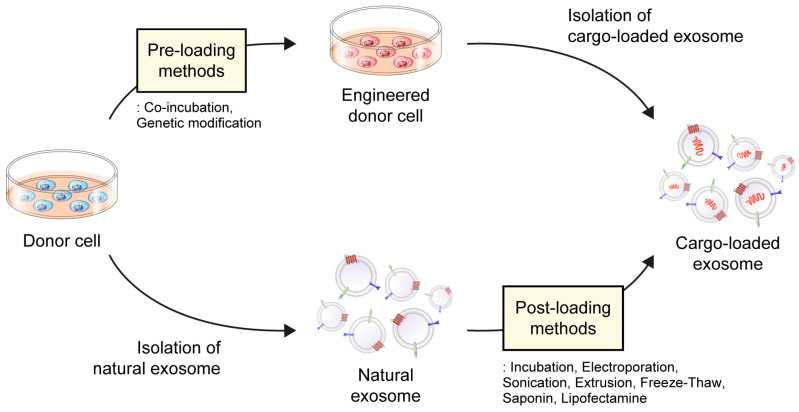
Drug-loading methods for exosome.

Drug-loading techniques can be classified into pre-loading (indirect-loading) and post-loading (direct-loading) methods (Figure 2) [51]. In the pre-loading technique, drugs are loaded into donor cells before exosome isolation, allowing for their encapsulation within exosomes during their natural biogenesis. The pre-loading method involves modifying donor cells by co-incubating them with the cargo or transfecting the target gene to generate targeted exosomes [52,53]. These modified cells are designed to produce and release exosomes containing specific cargo, such as drugs, proteins, and nucleic acids. Among them, it is advantageous to load proteins, siRNA, miRNA, and other drugs with high molecular weight [54,55]. The pre-loading method allows for the continuous production of cargo-loaded exosomes without compromising membrane integrity. However, its main limitation is the lack of precise control over the amount of cargo loaded. Additionally, this method exhibits low loading efficiency, may cause gene expression changes in donor cells, and has the potential for toxicity due to transfection agents [56]. In contrast, the post-loading method involves loading the drug onto pre-isolated natural exosomes. Within the post-loading approach, there are two distinct strategies, passive loading and active loading [55]. Passive loading relies on the physical properties of the drug to passively diffuse into exosomes, whereas active loading uses various strategies, such as electroporation, sonication, and extrusion, to encapsulate the drug into the exosomes [56,57]. In comparison to pre-loading, the post-loading method offers enhanced efficiency, as it allows for greater control over the encapsulation efficiency and loading capacity of the final product [58]. However, depending on the methods used, there could be limitations and risks to consider, such as exosome aggregation and potential membrane damage due to physical force, pH variation, and surfactants. Therefore, selecting the most suitable loading method is of utmost importance, considering the diverse technologies available that allow for tailoring the cargo content of exosomes to meet specific therapeutic needs.

### 4.1. Pre-Loading/Indirect Method

#### 4.1.1. Co-Incubation

Co-incubation is a process in which the cargo is loaded into donor cells and subsequently secreted in the form of exosomes. These exosomes can be isolated from the co-culture supernatants using ultracentrifugation. Although this method is relatively simple, it has limitations in terms of relatively low loading efficiency and the inability to precisely control the incorporation efficacy, which can be influenced by factors such as the physicochemical properties of the drugs and type of donor cell used. To improve loading efficiency, researchers have explored various techniques, including low-level electric treatment and ultraviolet irradiation [59,60]. A study examining the loading efficiency of doxorubicin across different cell types revealed that pancreatic cancer cells exhibit the highest loading efficiency, followed by macrophages and pancreatic stellate cells. Despite the lower loading efficiency of doxorubicin, exosomes derived from macrophages demonstrate greater antitumor activity than those derived from pancreatic cancer cells. These findings emphasize the variable efficiency of cargo loading depending on the specific type of parent cells involved and underscore the importance of considering the potential contributions of biomolecules when selecting suitable parental cells for co-incubation [61]. Finally, it is important to acknowledge the potential toxicity of the drug to parent cells. This can be assessed using techniques such as the 2,5-diphenyl-2H-tetrazolium bromide (MTT) assay, a commonly used cytotoxicity test. By evaluating the viability of parent cells, the MTT assay provides information regarding the potential adverse effects of the drug on cells used for cargo loading during the co-incubation process [52].

#### 4.1.2. Genetic Modification

The genetic modification of exosomes involves engineering donor cells to produce modified exosomes with specific properties. Several techniques have been employed to achieve this, including the introduction of plasmids, miRNAs, or other nucleic acids into cells. Although this method offers higher molecular stability than other approaches, it is time-consuming and its efficiency varies. For example, in a study involving miR-146b, a plasmid encoding this miRNA was introduced into marrow stromal cells through electroporation. Donor cells were then cultured, and exosomes carrying modified miRNAs were isolated using a precipitation polymer. The isolated exosomes exhibited miR-146b enrichment and retained typical marrow stromal cell marker expression. When exosomes were directly injected into a rat model of primary brain tumors, a significant reduction in glioma xenograft growth was observed [62]. These results are consistent with the fact that miR-146b can inhibit tumor necrosis factor (TNF) receptor-associated factor 6 (TRAF6) and Interleukin-1 receptor-associated kinase 1 (IRAK1) expression and the association between reduced expression of miR-146b and breast cancer, glioma, and large B-cell lymphoma [63,64,65,66]. However, as it is recognized to act as an oncogene in certain other types of carcinomas, such as papillary thyroid carcinoma, gastric cancer, and colorectal cancer, careful consideration should be given to its individual application based on the specific type of cancer [67,68,69]. Additionally, technology has been developed to increase the loading efficiency of cargo proteins using optogenetically engineered exosomes [70]. Exosomes for protein loading via optically reversible protein–protein interaction (EXPLOR) technology use blue light illumination to induce reversible associations between cryptochrome 2 (CRY2) and CRY interacting basic helix loop helix 1 (CIBN), two light-responsive binding proteins fused to a cargo protein or an exosome membrane protein. This can be achieved by transfecting parent cells with plasmids encoding CRY2 fused to the cargo protein and CIBN fused to the transmembrane protein, CD9. Following the initiation of transient docking between the CRY2-conjugated cargo protein and CIBN on the exosomes under blue light illumination, the cargo protein is introduced into the exosomes via endogenous biogenesis. Subsequently, the illumination source is removed, causing separation from the CIBN, allowing the cargo to be released into the luminal space and effectively delivered to the cytoplasm. The EXPLOR technology, which enables reversible conjugation and dissociation between target proteins and membranes, provides a stable approach for controlling the efficiency of loading therapeutic cargo into exosomes.

### 4.2. Post-Loading/Direct Method

#### 4.2.1. Passive Loading

One simple approach for compacting drugs into exosomes is passive loading, specifically using the incubation method. This incubation method facilitates the incorporation of drugs into exosomes by utilizing the concentration gradient that occurs when the external drug concentration is high. Although this method offers the advantages of simplicity of implementation and nondestructive effects on exosome integrity, its applicability is primarily restricted to specific types of drugs. This is particularly advantageous for hydrophobic drugs that interact with the lipid surfaces of exosomes. Consequently, the loading capacity relies on both the hydrophobic properties of the cargo and incubation duration [71]. For example, the hydrophobic polyphenol compound curcumin and hydrophobic anticancer drug paclitaxel can be loaded into exosomes via simple incubation [72,73]. Exosomes also contain a hydrophilic core that can be loaded with hydrophilic drugs; however, they do not readily interact with the lipid bilayer of exosomes, and the effectiveness of incubation is limited. In such cases, a modification of the incubation process using active loading techniques may be necessary to achieve adequate drug loading for clinical use [74]. Additionally, achieving precise control over the loading process can be challenging, and factors such as pH can also have an impact on the loading efficacy. For example, because weak basic conditions promote the diffusion of hydrophilic compounds through the lipid bilayer of exosomes, a pH condition of 8.0 was established to load hydrophilic doxorubicin into exosomes derived from macrophages [75].

#### 4.2.2. Active Loading

##### Physical Induction

To overcome the inherent limitations of passive loading, researchers have made significant advancements in the development of active cargo-loading techniques. Physical induction involves the use of external stimuli to facilitate the incorporation of specific cargo into exosomes. Various techniques have been employed, including electroporation, sonication, freeze–thawing, and extrusion. These methods induce transient pores or mechanical stress on the exosome membrane, allowing the efficient loading of cargo molecules [76]. These methods enable the loading of a wide range of cargoes into exosomes. The versatility of these loading methods provides options for the delivery of various therapeutic payloads, thereby broadening the potential applications of exosomes. Physical induction methods are relatively simple and can easily be implemented in laboratory settings. They offer precise control over the loading process, allowing the optimization of loading conditions and achieving the desired cargo loading levels. Although physical induction techniques offer numerous advantages, some challenges need to be addressed. The optimization of loading conditions and cargo compatibility with exosome membranes are critical for maintaining cargo stability and preventing cargo leakage during loading and subsequent delivery. Additionally, they can affect the targeting properties of exosomes by altering their intrinsic structure.

**Electroporation**

Electroporation is a commonly used, simple, and time-saving method for loading large molecules, such as miRNA and siRNA, into exosomes. This involves the application of an external electric field to exosomes that exceeds the energy that their phospholipid bilayer can withstand. This process leads to the formation of temporary pores in the exosomal membrane, resulting in instantaneous changes in cell membrane permeability [77]. When exosomes and drugs are exposed to this high-intensity electric field, drug molecules can enter the exosomes through pores created in the membrane [77]. After loading, the membrane returns to its original state. Electroporation is more efficient than passive co-incubation. When miRNA (cel-39) was loaded into plasma-derived EVs, the electroporation method (at 750 V and 10 pulses) demonstrated higher loading efficacy (31.9%) than direct incubation (7.9%). Additionally, electroporation provides superior protection against ribonuclease (RNase) degradation to incubation, with miRNA preservation rates of approximately 70% and 25%, respectively [78]. Unfortunately, the electroporation method has a drawback in that it can lead to the deformation or destruction of the membrane structure of the exosomes. This can be inferred from the changes in the zeta potential and colloidal stability of the exosomes after electroporation. However, in certain research settings, electroporation maintains the integrity of the vesicle cargo, including RNA and proteins, suggesting no vesicle damage. Therefore, carefully optimizing electroporation conditions to minimize changes in exosome structure and function during loading is important [78]. Electroporation potentially induces RNA aggregation, leading to a reduced loading capacity. To overcome this issue, a method of adding Ethylenediaminetetraacetic acid (EDTA) buffer to electroporation buffer can be used and, in this case, approximately 30% efficiency was achieved [79].

**Sonication**

After applying the sonication method, which utilizes sound energy, most MVs undergo a reorganization of their membranes [80]. This reorganization leads to a decrease in membrane microviscosity by creating pores on the membranes. Thus, disrupted membrane integrity allows the diffusion of hydrophilic or hydrophobic molecules or bioactive chemicals across the lipid bilayers. As a result, in many cases, the sonication method produces a higher loading efficiency than co-incubation and electroporation. In a study on loading paclitaxel into macrophage-derived exosomes, the loading capacity of sonication reached 28.3%, whereas alternative methods, such as incubation and electroporation, only yielded loading capacities of 1.4% and 5.3%, respectively [81]. Similarly, in a study investigating the loading of gemcitabine, sonicated exosomes demonstrated higher loading efficiency (11.7 ± 3.7%) than simply incubated exosomes (2.8 ± 0.7%). On the one hand, a concern is the possibility that sonication would irreversibly disrupt the integrity of lipid-based EVs, as sonication is often used as a means to disrupt lipids or cell membranes. On the other hand, during the process of loading paclitaxel into exosomes using mild sonication, exosome-bound proteins, including Alix, Tumor susceptibility gene (TSG) 101, and Flotillin, and the zeta potential remained unchanged. This indicates that mild sonication preserves the integrity of the exosome structure, including membrane-bound proteins and lipid components. Indeed, after the sonication procedure, complete restoration of membrane microviscosity was observed following a 1 h incubation, suggesting that the effects of sonication on the membrane structure are reversible depending on the conditions. Additionally, a study in which catalase was loaded into exosomes by sonication showed high loading efficiency and preservation of catalase enzyme activity against protease degradation, as well as long-term and sustained release [82].

**Extrusion**

The extrusion technique involves the extrusion of exosomes together with therapeutic cargoes through a membrane filter with a pore size of 100–400 nm [83]. The exosome-cargo mixture is repeatedly forced through the pores of the membrane under controlled pressure. This process facilitates the physical entrapment of therapeutic cargo within exosomes. Owing to its principal nature, extrusion helps to homogenize the size distribution of exosomes [80]. Moreover, the extrusion technique is effective for incorporating cargo into exosomes. Extrusion (22.2 ± 3.1%) and sonication (26.1 ± 1.2%) demonstrated significantly higher efficiency in enzyme incorporation than incubation (4.9 ± 0.5%), freeze–thaw (14.7 ± 1.1%), and incubation with saponin (18.5 ± 1.3%). These findings provide evidence for the superior performance of the extrusion method in loading therapeutic cargo into exosomes [82]. However, repeated extrusion raises concerns that exosomes and their cargo may be damaged by excessive shear stress, resulting in altered membrane properties [80]. Nevertheless, in certain settings, membrane composition or orientation may remain unaffected, which is supported by the maintenance of the zeta potential and membrane protein profile, albeit with a reduced size, after extrusion [84].

**Freeze–Thaw**

The freeze–thaw method disrupts the plasma membrane through mechanical stress induced by the formation and melting of ice crystals during freezing and thawing cycles. Disruption of the plasma membrane through the transient formation of ice crystals allows water-soluble molecules to be incorporated into the aqueous phase inside vesicles [85]. Exosomes are incubated with cargo at room temperature for an asset period and then frozen at −80 °C or in liquid nitrogen. The mixture is then thawed at room temperature. The freeze–thaw technique provides an intermediate loading efficiency for cargo into exosomes compared with sonication and extrusion [82]. Three or more freeze–thaw cycles are usually performed to improve drug encapsulation. However, as the number of freeze–thaw cycles increases, the concentration of exosomes and levels of their membrane proteins decrease [86,87]. Moreover, higher temperatures can affect the membranes of exosomes, altering their properties and facilitating their uptake by recipient cells. Therefore, identifying the appropriate temperature and cycle settings for optimal results is critical. Furthermore, the freeze–thaw method can be used to generate hybrid exosomes via the fusion of exosomes and liposomes. By manipulating the membrane lipid composition using liposomes via the freeze–thaw method, engineered polyethylene glycol-embedded exosomes exhibited a significant increase in cellular uptake compared with those that had not undergone freeze–thaw treatment [88].

##### Chemical Induction

**Saponin**

Saponins interact with cholesterol in cell membranes, facilitating the formation of pores in exosomes without compromising the integrity of the lipid bilayer structure. This property of saponins has been utilized to enhance the loading capacity of therapeutic cargo into exosomes, surpassing simple incubation methods. A previous study compared the efficacy of loading doxorubicin into EVs isolated from U937 cells using saponins, incubation at different temperatures, and freeze–thaw methods. The highest loading efficiency was observed when 0.2% saponins were added. Notably, only 20% of the total drug was released after 36 h, indicating sustained release. This sustained-release profile has the potential to mitigate off-target effects, as EVs tend to accumulate at the target site and, subsequently, release the therapeutic agent extracellularly [89]. Additionally, saponins are effective in loading hydrophilic molecules into exosomes. In particular, the saponin-assisted method has demonstrated remarkable results in active encapsulation techniques, achieving up to 11-fold higher loadings of hydrophilic porphyrins than passive methods [90]. However, there are concerns about saponin-related hemolytic activity in vitro; therefore, limiting the concentration of saponin used for drug loading and purifying exosomes after incubation with saponin are important [91].

**Lipofectamine**

Lipofectamine, a transfection reagent commonly used across diverse cell lines, significantly improves the transfection efficiency of different RNA molecules (such as mRNA and siRNA) and plasmid DNA in vitro. By combining nucleic acids with Lipofectamine, complexes are formed in which nucleic acids are encapsulated within lipid micelles. These complexes can then be introduced into exosomes and incubated [92]. One notable drawback of this method is the inability to separate exosomes from Lipofectamine, which raises uncertainty regarding whether the observed transfection can be attributed to Lipofectamine or the exosomes themselves.

## 5. Exosome Engineering

### 5.1. Genetic Engineering

Recently, researchers have actively explored strategies to enhance the targeting capabilities of exosomes. Genetic modification is a widely used technique that enables the incorporation of specific biomolecules onto the surface or within the lumen of exosome-producing cells. The surface of exosomes can be functionalized using ligands or peptides that enable recognition and binding to specific target cells or receptors. The molecular domains within the exosome membrane play a role in anchoring and presenting the desired biomolecules. Among these domains, non-specific membrane proteins, such as lysosomal-associated membrane protein 2 B (LAMP-2B) and the tetraspanins protein family, including CD63, CD9, and CD81, have been widely utilized as fusion partners for biomolecule attachment to exosomes [93]. Cell-specific receptor membrane proteins, such as epidermal growth factor receptor, platelet-derived growth factor receptor, human epidermal growth factor receptor 2 (HER2), and glycosylphosphatidylinositol, are also utilized for protein fusion and modification [94].

Various bioactive ligands can be conjugated to exosomes to enhance their targeting ability. Peptides characterized by their small size and relatively low immunogenicity can be used to enhance drug accumulation at specific sites of interest. For example, exosomal LAMP-2B can be genetically modified to incorporate the receptor for advanced glycation end-products (RAGE)-binding peptide (RBP), an anti-inflammatory peptide. These engineered exosomes can then be loaded with curcumin, thereby augmenting their anti-inflammatory properties and therapeutic potential [95]. RAGE, known for its high expression in the lungs and involvement in inflammatory responses in acute lung injury, serves as the target receptor [96,97]. In an acute lung injury model, after intratracheal administration, curcumin-loaded RBP-exosomes effectively reduce pro-inflammatory cytokine levels, inhibit inflammatory reactions, and co-localize with type I epithelial cells. These findings indicate the potential use of RBP exosomes as a therapeutic strategy for acute lung injury. In another study, HEK293T cells were genetically modified to produce exosome expressing LAMP-2B fused to a fragment of interleukin-3 (IL-3) [71]. As the IL-3 receptor is overexpressed in chronic myelogenous leukemia (CML) and acute myeloid leukemia blasts [98,99], exosomes derived from these cells loaded with imatinib or BCR-ABL siRNA effectively target and inhibit the growth of CML cells both in vitro and in vivo. This genetic engineering approach demonstrates the potential of utilizing IL-3-expressing exosomes as targeted drug delivery systems to overcome drug resistance in CML. Recently, a novel peptide sequence, the ischemic myocardial-targeting peptide CSTSMLKAC (IMTP), which can preferentially target the ischemic region of the heart, was discovered via in vivo phage display technology [100]. To harness this targeting potential, a research team fused IMTP with exosomal LAMP-2B using techniques, such as molecular cloning and lentiviral packaging, to generate IMTP-exosomes. IMTP-exosomes demonstrated a notable increase in accumulation, specifically within the ischemic heart area, compared with blank exosomes. These findings highlight the potential of IMTP-exosomes as targeted delivery systems for ischemic cardiac therapy [101]. Unlike other organs accessible through blood circulation, the efficient delivery of cargo to chondrocytes, situated within the dense and nonvascular extracellular matrix of cartilage, poses a significant challenge. To overcome this problem, chondrocyte-specific targeting was achieved by incorporating a chondrocyte-affinity peptide (CAP) into LAMP-2B on the exosome surface. With this modification, CAP-exosomes successfully encapsulated miR-140 and selectively entered chondrocytes in vitro, enabling the targeted delivery of the therapeutic cargo. Indeed, CAP-exosomes exhibited the remarkable ability to cross the dense mesochondrium, facilitating the delivery of miR-140 to deep cartilage sites and alleviating osteoarthritis progression in a rat model [102].

Antibodies can be used to improve the targeting capabilities of exosomes. For example, exosomes derived from A33-positive human colorectal cells (A33-Exo) were isolated and loaded with doxorubicin (A33-Exo/Dox). To specifically target A33-positive colorectal cancer cells, surface carboxyl superparamagnetic iron oxide nanoparticles (US) were coated with A33 antibodies (A33Ab-US) that bind to A33-positive exosomes to form a complex known as A33Ab-US-Exo/Dox. The complex exhibited a strong binding affinity and an enhanced anti-proliferative effect in colorectal cells. In a tumor-bearing mouse model, A33Ab-US-Exo/Dox displayed excellent tumor-targeting capability, effectively inhibiting tumor growth and prolonging the survival of mice, while minimizing cardiotoxicity [103]. However, full-length antibodies have limitations related to their large size, complex structure, and immunogenicity. Simplified antibody fragments, such as single-domain antibodies and single-chain variable fragments (scFvs), can also be used to improve the targeting ability of exosomes, while compensating for these limitations. In one study, HEK293 cells were genetically engineered to generate scFv domains that displayed an affinity for the cell surface receptor HER2. These scFv domains were fused with the C1C2 domain of lactadherin, which is highly abundant in exosome fractions. The uptake of these modified exosomes by breast cancer cell lines was significantly enhanced when the expression of HER2 in the recipient cells exceeded a specific threshold [104].

### 5.2. Chemical Engineering

The genetic engineering discussed has limitations in terms of cost and complexity and cannot be applied to pre-isolated exosomes. Alternatively, chemical engineering methods can be employed to directly attach molecules to the surface of exosomes via covalent bonds. Copper-catalyzed azide-alkyne cycloaddition, commonly referred to as click chemistry, is frequently employed for the conjugation of molecules to the surfaces of exosomes. In one particular study, the impact of the surface charge of exosomes on cellular uptake was explored by modifying the primary amine groups on the exosome surface using citraconic anhydride via Michael addition chemistry. The resulting modified exosomes, called cit-EXOs, exhibited a significantly reduced surface charge of approximately −50 mV compared with the control exosomes, whereas their hydrodynamic size remained similar. This study examined the intracellular uptake of these exosomes by two cell types: macrophages (RAW264.7) and dendritic cells (DC2.4). In the absence of serum proteins, the control exosomes and cit-EXOs demonstrated comparable levels of cellular uptake. However, in the presence of serum proteins, cit-EXOs exhibited superior uptake, specifically by RAW264.7 cells. These findings suggest that the chemical modification of exosomes via a reaction with citraconic anhydride holds promise as a strategy for engineering carriers that can be specifically targeted to macrophages [105]. Another research team connected a cyclo(Arg-Gly-Asp-D-Tyr-Lys) peptide (c[RGDyK]) to the exosomal membrane using click chemistry. c(RGDyK) has strong affinity to integrin αvβ3, which is found in cerebral vascular endothelial cells following an ischemic event. The intravenous injection of cRGD-exosomes (cRGD/Exo) efficiently targeted the affected areas within the ischemic brain. These exosomes successfully penetrated microglia, neurons, and astrocytes, indicating their ability to deliver therapeutic cargo to these crucial cell types at the site of brain lesions [106].

Carbodiimide chemistry involves the activation of the terminal carboxylic acid of a protein or phospholipid of exosomes, allowing direct conjugation to a primary amine through the formation of an amide linkage [107]. A carbodiimide species commonly used for this purpose is 1-ethyl-3-(3-dimethylaminopropyl) carbodiimide hydrochloride (EDC), which serves as a cross-linking reagent for carboxylic acids and primary amines. In one study, a cell-penetrating peptide, polyarginine, was conjugated to the surface of exosomes derived from HepG2 cells via an EDC/N-hydroxysuccinimide-mediated amide reaction. This conjugation approach not only enhanced the penetration capability of exosomes, but also facilitated the loading of antisense oligonucleotides into exosomes. Another class of chemical antibodies, nucleic acid aptamers, which consist of synthetic single-stranded DNA or RNA molecules with high target affinity, is also utilized for the surface modification of exosomes to enable targeted delivery. Their advantages include compact size, low immunogenicity, and straightforward chemical modification [108]. In a previous study, the 5TR1 aptamer, known for its specific recognition of the transmembrane mucin glycoprotein (MUC1) in cancer cells, was used as the targeting ligand. Doxorubicin was loaded into MSC-derived exosomes using electroporation. Subsequently, the surface amine functionalities of the exosomes were covalently conjugated to a MUC1 aptamer (apt-Exo/Dox). In an in vivo study using a mouse colon adenocarcinoma model (C26 ectopic model in BALB/c mice), apt-Exo/Dox significantly suppressed tumor growth compared with free doxorubicin. Furthermore, biodistribution analysis indicated a favorable distribution pattern with high accumulation in the tumor and faster clearance from the liver [109]. In another study, aptamer sgc8, known for its specific recognition of membrane-expressed protein tyrosine kinase 7, was utilized as a targeting ligand. To facilitate attachment to exosomes, the aptamer was conjugated to a diacyllipid tail via a polyethylene glycol (PEG) linker. This aptamer-PEG-diacyllipid complex was then introduced to the surface of the exosomes via hydrophobic interactions between the diacyllipid tail and exosomal phospholipid bilayer. The conjugation process involved simple mixing of exosomes with the sgc8-diacyllipid conjugate at 37 °C for 30 min, followed by ultracentrifugation. Additionally, doxorubicin was loaded into the engineered exosomes (sgc8-Exo/Dox). In vitro studies using a T-leukemia cell line demonstrated specific recognition of target cells by sgc8-Exo. Moreover, the therapeutic efficacy of sgc8-Exo/Dox was observed to be superior to that of free doxorubicin, indicating enhanced cellular accumulation of the therapeutic cargo [110].

## 6. Clinical Application of Exosomes-Based Drug Delivery

### 6.1. Cancer

**Figure 3:**
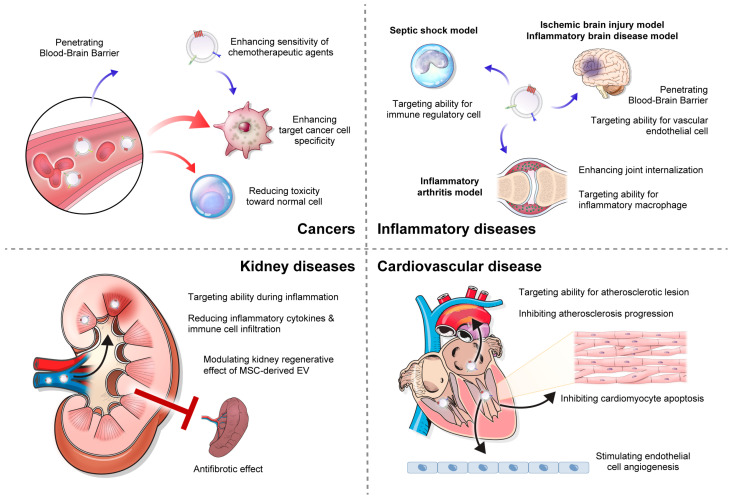
Clinical application of exosome-based drug delivery.

Despite significant advancements in anticancer drugs, many current cancer therapies still lack specificity for targeting cancer cells, leading to high toxicity toward normal cells. Moreover, poor drug absorption hampers treatment efficacy, necessitating high drug concentrations. To address these issues, exosome-based drug delivery has emerged as a promising strategy for drug delivery in cancer treatment (Figure 3). Exosomes serve as effective carriers for a wide range of cancer therapeutic cargoes, including anti-cancer small-molecule drugs, nucleic acids, and proteins, enabling safe delivery and sustained release.

#### 6.1.1. Small-Molecule Drugs

Paclitaxel is an antineoplastic agent belonging to a class of drugs known as taxanes. The anti-tumor effect of paclitaxel is mediated by its ability to stabilize microtubules and suppress cell division, leading to cancer cell death [111,112]. It is used to treat a broad spectrum of cancers, including glioblastoma multiforme and breast, ovarian, lung, and pancreatic cancers. However, the low solubility and dose-dependent toxicity of paclitaxel pose clinical obstacles to its medical use. Research on paclitaxel delivery using exosomes was first conducted using mesenchymal stromal cells. Mesenchymal stromal cells primed with paclitaxel produce paclitaxel-loaded exosomes that subsequently exhibit enhanced anti-tumor effects [52]. Further studies have shown the efficient transportation of paclitaxel by various types of exosomes. In one study, the utilization of bovine colostrum-derived exosomes as carriers for paclitaxel delivery showed anti-tumor effects in lung cancer [113]. Another study supported the role of M1 macrophage exosomes as paclitaxel transporters, augmenting the anti-tumor effects of chemotherapy in mice with tumors [114]. The use of exosomes derived from malignant glioma cells (U-87) allows paclitaxel to penetrate the blood–brain barrier, offering a potential therapeutic strategy for glioblastoma multiforme [73].

Doxorubicin is an effective antitumor agent against leukemia, Hodgkin lymphoma, and solid tumors. However, its continuous chemotherapeutic use is limited by low bioavailability and side effects, such as cardiotoxicity and bone marrow suppression [115]. Exosomal doxorubicin has the advantage of mitigating heart toxicity by partially reducing the delivery of doxorubicin to myocardial endothelial cells. As a result, higher concentrations of doxorubicin can be administered, leading to enhanced efficacy against breast and ovarian tumors in mice [116]. Furthermore, recent studies have focused not only on incorporating doxorubicin into exosomes, but also improving its binding properties via the expression of disintegrins and metalloproteinase 15 on exosomal membranes. The results revealed improved targeting properties and production yield, consequently exhibiting improved antitumor effects in triple-negative breast cancer, without adverse effects [117]. Other researchers enhanced the delivery of doxorubicin to brain tumors by utilizing neutrophil-derived exosomes. Given a previous finding indicating the ability of neutrophils to infiltrate inflamed brain tumors, neutrophil-derived exosomes (NE-Exos) were chosen for their potential to penetrate the blood–brain barrier (BBB) and target tumors. Subsequently, doxorubicin and NE-Exo were combined and sonicated to form DOX-loaded NE-Exo (NE-Exo/DOX). NE-Exo/DOX efficiently penetrated the BBB and migrated to the brain in vivo in zebrafish and C6-Luc glioma-bearing mouse models, demonstrating their ability to facilitate drug delivery. Moreover, in a glioma mouse model, the intravenous injection of NE-Exo/DOX demonstrated remarkable efficiency in suppressing tumor growth and significantly prolonging survival time [118].

Curcumin, a naturally occurring polyphenol compound, inhibits the initiation and metastasis of various types of cancers. However, its clinical use is limited owing to poor absorption and rapid metabolism. To overcome these limitations and safely deliver curcumin to the target cells, researchers have explored the use of curcumin-loaded exosomes. Owing to its hydrophobic nature, curcumin shows a fivefold increase in solubility when incorporated into PBS-containing exosomes compared with PBS without exosomes. Additionally, exosome-loaded curcumin exhibits significantly higher stability, with over 80% remaining after a 150 min incubation in PBS at pH 7.4, than free curcumin, which rapidly degraded and only retained 25% of its initial concentration. Furthermore, the in vivo administration of exosomal curcumin via intraperitoneal or oral routes results in a five- to tenfold higher accumulation of curcumin in the peripheral blood than that of curcumin alone [72]. In a study conducted on nude mice implanted with cervical CaSki tumor xenografts, the delivery of curcumin through curcumin-loaded exosomes resulted in higher concentrations of curcumin in the blood than the dietary route. Consequently, the group that received curcumin-loaded exosomes showed significant inhibition of cervical tumor xenografts without any evidence of systemic toxicity [119]. The utilization of exosomes for the safe delivery and enhanced bioavailability of curcumin has demonstrated their potential to treat various types of cancers, including breast cancer, glioblastoma, and lung cancer [120,121,122,123,124].

#### 6.1.2. Therapeutic Nucleic Acids

Gene therapy has emerged as a promising approach for cancer treatment that utilizes cell manipulation to exert therapeutic effects by delivering nucleic acids as therapeutic agents. This innovative therapy aims to address aberrant gene expression by editing the genome or introducing exogenous genes that interfere with the translation of faulty mRNA. Viral vectors have been widely used for nucleic acid delivery in gene therapy. However, their use is accompanied by challenges, including potential toxicity and immune responses [125]. Non-viral vectors, although alternatives, have limitations, such as low transduction efficiency and large particle sizes, which can trigger immune reactions and result in rapid clearance by the reticuloendothelial system [126]. Consequently, the clinical application of gene transfer vectors necessitates the development of non-immunogenic, tissue-specific, and non-toxic vectors to overcome these limitations, and exosomes have emerged as a promising solution (Table 1).

miRNAs are a class of small noncoding RNAs that play a crucial role in regulating gene expression by binding to the 3’ untranslated regions of target mRNAs. This interaction leads to mRNA degradation or translational repression, thereby modulating the expression of genes involved in various cellular processes relevant to cancer progression. miRNA-122 plays a crucial role in various aspects of liver physiology and pathology. The loss or downregulation of miR-122 has been strongly linked to the development, progression, and metastasis of hepatocellular carcinoma [127]. Based on this theoretical foundation, a study group transfected adipose tissue-derived MSCs (AMSCs) with a plasmid encoding miR-122. Subsequently, miR-122 was delivered to hepatocellular carcinoma cells through AMSC-derived exosomes, potentially enhancing the sensitivity of these cells to chemotherapeutic agents by negatively regulating the expression of miR-122 target genes. The inhibitory effects of 5-FU and sorafenib on hepatocellular carcinoma cells treated with miR-122-loaded exosomes were significantly enhanced compared with controls treated with exosomes lacking miR-122 [128]. Another study provided evidence for the potential of miRNA-126 as a treatment for lung cancer. This study also evaluated the concept of organotropism in exosomes by utilizing breast cancer cell-derived exosomes (231-Exo) as targeted carriers for non-small-cell lung cancer cells. Their approach was based on the identification of integrin β4 overexpression in breast cancer cell-derived exosomes, which interacts with surfactant protein C on the surface of lung cancer cells. This interaction demonstrated the remarkable ability of 231-Exo to specifically recognize and target lung cancer cells circulating in the bloodstream, effectively evading immune surveillance in vitro. To determine the therapeutic potential of these exosomes, they were loaded with miRNA-126 to create miRNA-126-loaded 231-Exo (miRNA-126/231-Exo). Intriguingly, the intravenous administration of miRNA-126/231-Exo resulted in efficient homing to the lung region in an in vivo mouse model. Moreover, in a lung metastasis model, miRNA-126/231-Exo demonstrated outstanding efficacy in inhibiting lung metastases. The underlying mechanism by which miRNA-126/231-Exo exerts its inhibitory effects on lung cancer cells is via the disruption of the PTEN/PI3K/AKT signaling pathway. This disruption leads to the suppression of lung cancer cell proliferation and migration, contributing to the overall antimetastatic effects observed [129].

Small interfering RNAs (siRNAs) modulate gene expression via RNA interference. This technology has great potential for overcoming drug resistance in various cancers. However, siRNAs have low stability and a tendency to rapidly degrade in the systemic circulation. To address this challenge, the use of exosomes as delivery vehicles for siRNAs has garnered significant attention and shown considerable promise. In a study using a CML model, BCR-ABL siRNA was delivered to CML cells via engineered exosomes. Exploiting the fact that the IL3 receptor is overexpressed in CML cells compared with normal cells, IL3-LAMP-2B-expressing exosomes specifically targeted cells. These exosomes were loaded with either imatinib or BCR-ABL siRNA and successfully delivered to CML cells to inhibit cancer cell growth. Treatment with BCR-ABL siRNA-loaded exosomes significantly reduced BCR-ABL expression at both mRNA and protein levels [71]. Similarly, exosomes loaded with carnitine palmitoyltransferase 1A siRNA inhibit fatty acid oxidation, a crucial factor in drug resistance in cancer cells, and effectively reverse oxaliplatin resistance in colon cancer [130]. These experiments also involved the modification of exosome membranes using iRGD peptides, which can enhance extravasation and targeted infiltration by interacting with integrin αvβ3 or αvβ5 and neuropilin-1 present on tumor vascular endothelium and tumor cells. Exosomes delivering c-Met siRNA effectively decrease c-Met expression in gastric cancer cells and successfully reverse cisplatin resistance [131].

**Table 1:** Application of exosome-based therapeutic nucleic acids delivery in cancer.

Nucleic Acid	Cellular Source of Exosome	Transfection Method	Target Disease [Animal Model]	Mechanism	Refs
hsa-miR-122	Human adipose tissue-derived mesenchymal stem cell	Lipofectamine	Hepatocellular carcinma [BALB/c nude mice + HepG2 cells]	Enhancing sensitivity of chemotherapeutic agents (5-fluorouracil and sorafenib) through altering target genes expression	[128]
miRNA-126	Human breast cancer cell	ExoFectin	Lung metastasis model [C57BL/6 + A549 cells]	Interaction between surfactant protein C on cancer cell and integrin β_4_ on exosome Inhibiting proliferation and migration of cancer cells via a PTEN/PI3K/AKT signaling pathway	[129]
BCR-ABL siRNA	HEK293T cell (engineered using IL3-LAMP-2B plasmid)	Lipofectamine	CML model [NOD/ SCID mice + human LAMA84 cells or K562R cells]	Interaction between IL-3 on exosome and IL3R on CML blast Reverse sensitivity of chemotherapeutic agent (Imatinib) through decreasing BCR-ABL mRNA and protein level	[71]
CPT1A siRNA	HEK293T cell (engineered using iRGD- LAMP-2B plasmid)	Lipofectamine	Colon cancer mocel [BALB/c nude mice + HCT116-lohp cell]	Interaction between iRGD on exosome and αvβ3 and Neuropilin-1 on colon cancer cell Reverse the sensitivity of chemotherapeutic agent (oxaliplatin) through inhibiting fatty acid oxidation	[130]
BCL6 siRNA1	Mouse bone marrow dendritic cell (engineered using iRGD- LAMP-2B plasmid)	Electroporation	Diffuse large B-cell lymphoma (DLBCL) model [BALB/c nude mice + OCI-Ly8 cells]	Interaction between iRGD on exosome and αvβ3 integrin on DLBCL cell Inhibiting DLBCL cell proliferation through inducing G0-G1 cell cycle arrest	[132]
c-Met siRNA	HEK293T cell	Lipofectamine	Gastric cancer model [BALB/c-nu + SGC7901/DDP]	Enhancing sensitivity of chemotherapeutic agent (cisplatin) through altering target genes expression	[131]

### 6.2. Inflammatory Diseases

Numerous investigations have examined the impact of exosomes on various inflammatory conditions, such as sepsis, arthritis, inflammatory bowel disease, and neurodegenerative disorders [133,134,135,136,137]. These studies indicated that exosomes play a significant role in the progression of inflammatory diseases. Building on this understanding, there is growing interest in advancing the technology to encapsulate anti-inflammatory agents within exosomes that possess inherent or engineered tropism to specific sites of inflammation (Figure 3). This innovative approach aims to improve the targeted delivery of therapeutics and has great potential to effectively alleviate the inflammatory response, while reducing systemic side effects.

Curcumin, which has been studied for its application in cancer treatment, has also been extensively used for the management of inflammatory diseases. Its potent anti-inflammatory properties have garnered significant attention and led to its widespread use in various inflammatory conditions. CD11b+Gr-1+ myeloid-derived suppressor cells play pivotal roles in the pathogenesis of inflammatory diseases. Their accumulation can disrupt the host immune response and impair immune surveillance, thereby contributing to inflammation and tumor progression. In an experimental model of lipopolysaccharide (LPS)-induced septic shock, the administration of curcumin-loaded exosomes derived from a mouse lymphoma cell line caused notable benefits, including improved survival rates and reduced production of interleukin-6 (IL-6) and TNF-α compared with treatment with curcumin alone. These effects were attributed to the ability of exosomes to enhance the delivery of curcumin, specifically to CD11b+Gr-1+ cells, resulting in increased apoptosis. For these reasons, targeting CD11b+Gr-1+ cells with exosome-delivered curcumin offers a promising approach for treating inflammation-related diseases, including cancer, by addressing the underlying mechanisms associated with these conditions [72]. The same research group loaded curcumin into exosomes isolated from the same cell lines and tested its effects by intranasal administration in LPS-induced inflammatory brain disease and autoimmune encephalitis models. The intranasal administration of exosomes (30–100 nm) results in their translocation into the brain. In contrast, larger microparticles (500 nm–1 μm) are primarily delivered to the lungs and intestines, underscoring the importance of particle size in determining translocation across the BBB from the nasal region to the brain. The findings of this study demonstrated a significant decrease in the population of inflammatory microglia (CD45.2+IL-1β+) in the brains of mice treated with intranasal exosome-curcumin. Moreover, treatment delayed the progression of autoimmune encephalomyelitis induced by myelin oligodendrocyte glycoprotein peptides. Because microglia play a crucial role in various inflammatory brain diseases, the ability of exosomes to traverse the blood–brain barrier and target microglia highlights their potential as drug delivery systems for inflammatory brain diseases [138]. Another study investigated the anti-inflammatory effects of curcumin-loaded exosomes in a cerebral ischemia model. Exosomes were engineered using the c (RGDyK) peptide to enhance targeted delivery to vascular endothelial cells in the ischemic brain. Modified exosomes (cRGD/Exo/Cur) were administered intravenously. Curcumin-loaded engineered exosomes effectively alleviated inflammation and reduced apoptosis in ischemic brain regions. However, there was a potential concern regarding the accumulation of cRGD-Exo in other organs, particularly the liver, owing to the high levels of integrin αvβ3 found in liver tissue [106].

In arthritis research, researchers have explored methods for using glucocorticoids by loading them with exosomes. To achieve this, exosomes were isolated from RAW264.7, a macrophage cell line, and dexamethasone was loaded into them via electroporation. Additionally, to enhance the targeted delivery of the drug to folate receptor β, exosome membranes were engineered with folic acid-PEG-glycol-cholesterol. The comparative efficacy of engineered exosome-dexamethasone (FPC-Exo/Dex), exosome-dexamethasone (Exo/Dex), liposome-dexamethasone (Lip/Dex), and free dexamethasone (Dex) formulations was evaluated in a model of collagen-induced arthritis. FPC-Exo/Dex exhibited greater accumulation in the joints than the other dexamethasone formulations, indicating enhanced delivery at inflammatory sites. Additionally, FPC-Exo/Dex exhibited superior therapeutic effects to other dexamethasone formulations, resulting in reduced histopathology scores and less cartilage and bone destruction, which was potentially attributed to its enhanced targeting and internalization, along with the modulation of cytokine secretion. Loading glucocorticoids into exosomes is a promising approach to mitigate the serious side effects associated with the long-term use of systemic glucocorticoids, such as immunosuppression, hyperglycemia, and osteoporosis [139]. This delivery method offers the potential for improved therapeutic efficacy by specifically targeting drugs to inflamed tissues, thereby enhancing performance, while minimizing side effects [140]. In another study on rheumatoid arthritis, exosomes were utilized as carriers to deliver interleukin-10 (IL-10) cytokine-encoding plasmids (IL-10 pDNA) and betamethasone simultaneously. These exosomes were isolated from a murine macrophage cell line (RAW264.7) pretreated with interleukin-4 (IL-4) to promote M2 polarization. M2 exosomes exhibited increased expression of lymphocyte function-associated antigen 1 (LFA-1) and very late antigen 4 (VLA-4) on their surfaces, whereas their corresponding receptors were expressed inversely in LPS-activated macrophages. This molecular recognition between M2 exosomes and activated macrophages was indirectly confirmed by the distribution of M2 exosomes, which predominantly accumulated in the inflamed joints of a collagen-induced arthritis mouse model. Co-encapsulation of the plasmid and betamethasone into exosomes was achieved using incubation and electrophoresis methods, respectively. M2 exosome-pDNA-betamethasone (M2 Exo/pDNA/BSP) effectively increased IL-10 secretion and suppressed the production of pro-inflammatory cytokines of IL-1β and TNF-α, efficiently polarizing macrophages from the pro-inflammatory M1 state to the anti-inflammatory M2 state [141].

### 6.3. Kidney Diseases

Kidney disease represents a significant global health burden affecting millions of individuals worldwide [142,143,144,145]. Despite advancements in treatment, the management of acute kidney injury (AKI) is mostly conservative [146,147,148], and the treatment of glomerulonephritis with immunosuppressive agents is accompanied by systemic side effects [149,150,151]. A preclinical study has demonstrated the potential of exosome-mediated drug delivery in kidney diseases, including AKI and kidney fibrosis (Figure 3) [152].

Glucocorticoids are essential medications prescribed to treat kidney diseases associated with inflammation. However, the long-term use of systemic glucocorticoid therapy can lead to severe side effects, which worsen as the dosage increases. To overcome these challenges, researchers have explored the potential of using MVs, specifically focusing on their properties of membrane protein function preservation during budding from the plasma membrane and their isolation feasibility compared with exosomes. They treated RAW264.7 cells with dexamethasone, and MVs were isolated from the supernatants by sequential centrifugation. These macrophage-derived MVs exhibited enhanced accumulation in inflamed kidneys compared with healthy kidneys, indicating their improved targeting ability during inflammation. This targeting is mediated through the interaction of specific surface proteins, such as LFA-1 and VLA-4, on macrophage-derived microvesicles with intercellular adhesion molecule 1 (ICAM-1) and vascular cell adhesion molecule 1 (VCAM-1) in the kidney. In murine models of LPS- or Adriamycin-induced nephropathy, MVs loaded with dexamethasone (MV-DEX) showed superior therapeutic efficacy to free dexamethasone treatment. MV-DEX significantly attenuates renal injury and reduces inflammation and fibrosis. In vitro studies have demonstrated that MV-DEX inhibits NF-κB activity, achieving significant anti-inflammatory effects with only one-fifth of the required dose of free dexamethasone. The delivery of dexamethasone through MVs reduces the side effects associated with chronic glucocorticoid therapy, such as hyperglycemia and suppression of the hypothalamus–pituitary–adrenal axis [153]. Another research group utilized the super-repressor IĸB (srIkB), which impedes the nuclear translocation of NF-ĸB, in a kidney ischemia-reperfusion injury (IRI) mouse model. srlkB was encapsulated in optogenetically (EXPLOR) engineered exosomes (Exo/srIkB) [70]. In vivo imaging demonstrated that Exo/srIkB was taken up by neutrophils and macrophages in the IRI model, and flow cytometric analysis revealed a significant reduction in the frequency of neutrophils, monocytes, macrophages, and T cells in ischemic kidney tissue. Consequently, the systemic administration of Exo/srIkB effectively suppressed NF-kB signaling in post-ischemic kidneys. This results in the decreased expression of pro-inflammatory cytokines, chemokines, and adhesion molecules, along with the mitigation of apoptosis, ultimately leading to kidney function protection [154]. This immune cell-mediated kidney-protective effect of Exo/srIkB was also demonstrated in a mouse model of sepsis [155].

Exosome-loaded miRNAs have also been used to diagnose and treat kidney diseases [156]. In a previous study, potential therapeutic miRNAs were identified using RNA sequencing analysis in AKI mice [157]. One research group attempted to identify the kidney protective effects of loading these miRNAs into EVs [158]. MSCs were transfected with healing miRNA mimics (has-miR-127, has-miR-486, has-miR-10a, and has-miR-29a) via electroporation (990 V, 40 ms, 1 pulse). EVs from MSC-EPs transfected with 10a and 486 miRNA mimics induced the significant proliferation of murine tubular epithelial cells compared with negative controls. However, when they were applied to an AKI mice model, treatment with a dose of miRNA-enriched EVs equivalent to the effective dose of naïve EVs did not result in any significant improvement. Engineered EVs were more effective than naïve EVs at suboptimal doses. This finding suggests that the kidney regenerative effect of MSC-derived EVs is influenced by a balanced miRNA composition. Hence, modifying the miRNA content within EVs holds promise for enhancing the therapeutic efficacy of EVs in AKI, particularly at lower doses.

In another study, miRNA-29 was utilized as a therapeutic cargo loaded into exosomes derived from satellite cells in a mouse model of unilateral ureteral obstruction (UUO). miR-29 attenuates muscle wasting and exhibits antifibrotic activity in chronic kidney disease [159]. To engineer exosomes, LAMP-2B was fused with rabies virus glycoprotein peptide, a protein that targets organs that express acetylcholine receptors, such as the kidneys. Adenoviral vectors were then used to transduce miR-29aba and miR-29b2c into exosomes. The injection of Exo/miR-29 into the skeletal muscle demonstrated promising results in mitigating UUO-induced weight loss and muscle atrophy. Furthermore, the intramuscular administration of Exo/miR-29 slowed the progression of renal fibrosis. This effect was mediated by the downregulation of transcription factor Yin Yang 1 and transforming growth factor β3. These engineered exosomes have been demonstrated to deliver high concentrations of therapeutic miRNAs to the injured kidney, suggesting their potential to treat fibrotic lesions in the kidney [160].

### 6.4. Cardiovascular Diseases

Cardiovascular disease is the leading cause of morbidity and mortality worldwide. Despite significant advancements in therapeutic interventions, there is a pressing need for novel strategies to effectively target and treat cardiovascular pathologies. Decades of research have been dedicated to studying stem cell therapy as a means of restoring myocardial function. Among the various cell types explored, MSCs have emerged as promising therapeutic agents capable of restoring cardiac function via multifaceted mechanisms. Nonetheless, direct cell therapy approaches raise concerns owing to challenges such as low cell retention and survival, as well as potential issues related to immunogenicity and oncogenicity. To address these concerns, researchers have explored the use of exosomes loaded with therapeutic cargo (Figure 3).

miRNA-21 is a key player in inhibiting apoptosis and promoting angiogenesis in cardiovascular diseases [161]. It targets PDCD4/AP-1 to inhibit apoptosis in cardiomyocytes and activates PTEN/Akt signaling to stimulate angiogenesis in endothelial cells. Recently, researchers have successfully encapsulated miR21 in EVs derived from HEK293T cells. Approximately 47.2% of the miR21-EVs exhibited a size range of 30 to 150 nm, consistent with exosomal characteristics, as confirmed by the presence of exosomal markers CD9, CD63, and CD81. These miR21-loaded EVs (miR21-EVs) effectively reduce PDCD4 protein levels, an miR12 target gene, and suppress apoptosis in cardiomyocytes and endothelial cells. However, liposome-based delivery fails to achieve this effect. The intramyocardial injection of miR21-Evs demonstrated their distribution in the infarcted heart, and they showed effects that promoted cardiac function recovery [162].

Atherosclerosis, a chronic inflammatory disease characterized by the formation of plaques on arterial walls, is a significant contributor to cardiovascular diseases, such as coronary artery disease, stroke, and peripheral vascular disease. Although many treatments for atherosclerosis are currently employed in clinical practice, a considerable number of patients still face a high risk of cardiovascular disease, even with optimal treatment. Consequently, there is a need to identify new therapeutic targets and develop more effective treatment approaches to address this persistent challenge. To achieve this, researchers employed a strategy involving the transfection of miR-221, which plays an important role in the regulation of atherosclerotic plaque stability [163], into MSCs to generate EVs enriched with overexpressed miR-221, referred to as EV-miR-221. These EVs were then administered to atherosclerotic mice via intravenous injection over a 2-week period. Compared with that in atherosclerotic mice treated with a control substance (PBS), the delivery of miR-221 through EVs led to the downregulation of the expression of *N*-acetyltransferase-1, which is highly expressed in atherosclerotic lesions. Furthermore, this intervention effectively inhibited the formation of aortic plaques in atherosclerotic mice, demonstrating the potential of EV-mediated miR-221 delivery as a promising therapeutic approach to address atherosclerosis [164]. The therapeutic effects on atherosclerosis have also been evaluated by using inflammation-responsive IL-10 mRNA-loaded exosomes. As the internal ribosome entry site (IRES) of the hepatitis C virus (HCV) is enriched in miR-122, which is responsible for the specific translation of viral proteins in the liver, it can alter the conformation of IRES [165]. By replacing the recognition site of the HCV-IRES with a sequence targeted by miR-155, it is possible to create a functional mRNA that specifically responds to atherosclerotic lesions. IL-10 was engineered to harbor the modified HCV-IRES. This approach was based on previous findings showing elevated miR-155 levels in atherosclerotic plaques [166]. Engineered exosomes containing modified mRNA (Exo/IRES-IL10) were assessed in an Apolipoprotein E-deficient mouse model of atherosclerosis. These findings revealed the targeted delivery of exosomes to CD68+ cells, potentially macrophages, within the plaques, as confirmed by confocal microscopy. Furthermore, a 2-week administration of Exo/IRES-IL-10 resulted in fewer atherosclerotic plaques, with increased levels of Il-10 mRNA and a decrease in IL-1β, TNF-α, and IL-6 levels within the plaques [167].

### 6.5. Neurodegenerative Diseases

Alzheimer’s disease (AD) is the most common neurodegenerative disorder, marked by the accumulation of amyloid-β (Aβ) peptides, hyperphosphorylation of tau proteins, and loss of neurons and synapses. This leads to a gradual and progressive decline in cognitive functions [168]. Because of the complexity of AD, only a few drugs have been clinically administered to treat the condition. Therefore, there is an urgent demand to develop specialized and effective approaches for treating AD. In AD, the overexpression of β-site amyloid precursor protein (APP)-cleaving enzyme 1 (BACE1) promotes the enzymatic digestion of APP, resulting in amyloid plaque formation and the accumulation of Aβ, contributing to senile plaque formation [169]. In a study, the miR-29, a known for targeting BACE1, was explored as a potential therapeutic agent for AD. HEK-293T cells and rat bone marrow mesenchymal stem cells were transfected with recombinant expression vectors containing mir-29a or mir-29b precursor sequences. When isolated miR-29-loaded exosomes were administered into an amyloid-beta induced AD model, they prevented spatial learning and memory impairments. The mechanism of its therapeutic effect was supported by in vitro results showing effective downregulation of BACE1 in U87 glioblastoma cells treated with miR-29b-loaded exosomes. Another study has demonstrated the therapeutic effect of curcumin-loaded exosomes by inhibiting Tau hyperphosphorylation through the AKT/GSK-3β pathway [170]. In the okadaic acid-induced AD mouse model, exosome derived from RAW 264.7 macrophage cells were employed for targeted delivery of curcumin across the blood–brain barrier. These curcumin-loaded exosomes exhibited high expression of lymphocyte function-associated antigen 1 (LFA-1), facilitating interaction with ICAM-1, thereby leading to increased accumulation in the brain than other organs (brain: 6.5 folds, liver: 2.5 fold, and lung: 2.0 fold). Treatment with curcumin-loaded exosomes resulted in decreased expression of Bax and cleaved caspase-3, leading to increased neuronal cell viability in the CA1, CA3, and DG regions of the hippocampus.

Parkinson’s disease (PD) is the second most common neurodegenerative disease, marked by the progressive degeneration of dopaminergic neurons in the substantia nigra. The molecular pathogenesis of PD involves a various pathways and mechanisms, including α-synuclein proteostasis, mitochondrial function, neuroinflammation, and oxidative stress [171]. It has been demonstrated that exosomes secreted from macrophages can promote drug delivery to neurovascular target cells, such as neurons, astrocytes, and brain microvascular endothelial cells [172]. In a study, researchers utilized exosomes derived from mouse macrophage cell line RAW 264.7 and loaded them with catalase (Exo/CAT) [82]. In the PD mouse model, the intranasal administration of Exo/CAT resulted in a widespread distribution of exosomes throughout the brain, with a notable presence in various regions, including the cerebral prefrontal cortex, central sulcus, and cerebellum. This treatment exhibited an anti-inflammatory effect, evidenced by significant reductions in microgliosis and astrocytosis, as indicated by lower levels of CD11b and glial fibrillary acidic protein. Additionally, there was a remarkable increase in the survival rate of dopaminergic neurons. Another study group investigated the application of blood-derived exosomes in PD mouse models to enhance BBB penetration. Since a substantial number of exosomes are released during the maturation process of reticulocytes into erythrocyte vesicles, they possess a significant advantage in terms of easy acquisition [173]. Following isolation from mouse blood, exosomes were incubated with dopamine, and subsequently purified as dopamine-loaded exosomes (Exo/Dopa) through ultracentrifugation. The administration of Exo/Dopa resulted in improved behavioral imbalance and an increase in the levels of dopamine, tyrosine hydroxylase, and antioxidant capacity within the lesioned striatum. The therapeutic dosage of Exo/Dopa demonstrated no toxic effects on the heart, liver, or kidney when compared to free dopamine, suggesting that blood exosomes hold promise as a safe and targeted drug delivery system for the PD.

## 7. Challenges Associated with Exosome-Based Drug Delivery and Future Perspectives

Exosome-based drug delivery technology is promising in the pharmaceutical field, offering notable advantages, such as enhanced bioavailability in circulation, targeted delivery capabilities, and reduced immune reactions. However, several limitations need to be addressed before entry into the clinical field. Foremost, there have been substantial improvements in isolation and purification techniques to overcome the low efficiency and heterogeneity of exosome products. Fortunately, isolating exosomes from the supernatant of cell cultures is easier than isolating exosomes from body fluids, such as plasma, saliva, and urine [174]. However, exosomes derived from cell supernatants may also contain other EVs, such as MVs, apoptotic bodies, and non-vesicular contaminants. Commonly used cost-effective and automated ultracentrifugation has limitations, such as difficulty in distinguishing exosomes from other EVs within the same size range and variability in yield efficiency. Alternatively, immunoaffinity capture can be used to isolate specific antigen-containing exosomes; however, there is no unique marker for exosomes, and it is not suitable for mass production in clinical applications. Other methods, such as density gradient centrifugation, ultrafiltration, and polymer-based precipitation, are also available; however, it is difficult to choose a single method and claim that it is the gold standard. Therefore, it is necessary to establish individualized isolation and purification methods. A comprehensive understanding of the advantages and disadvantages of different exosome production and preparation methods can aid in selecting the most suitable approach for exosome synthesis. Additionally, there are possible optional technologies for enhancing the production yield of exosomes, such as genetic manipulation and different controls over cell culture conditions. For example, the upregulation of Hsp20 in cardiomyocytes has been shown to enhance exosome secretion by directly interacting with Tsg101, an initiator of the exosome biosynthetic pathway [175]. The inhibition of PIKfyve, a phosphoinositide kinase, increases exosome secretion by inducing secretory autophagy [176]. Elevated intracellular calcium levels stimulate an increase in exosome release, which is mediated by the Ca^2+^-dependent Rab-binding protein Munc13-4 [177]. Based on these theoretical studies, if the drug-loading technology is genetically or chemically manipulated, it is possible to produce exosomes in high yields. Additionally, the effects of natural exosomes should be considered. Exosomes inherently have a biological function because they carry internal contents, such as DNA, RNA, and proteins, rather than simply serving as empty carriers. Furthermore, the structure and function of exosomes vary depending on the cell source, culture conditions, and engineering techniques employed. Therefore, it is very important to find a cell source that is well-suited for the clinical use of exosomes. Tumor cell-derived exosomes or immortalized cell lines are often utilized in many studies aimed at enhancing the targeting efficiency of cancer cells; however, it is essential to consider the potential oncogenicity associated with their use [178]. Alternatively, exosomes derived from red blood cells have been employed to deliver nucleic acid-based therapeutics, offering the advantage of originating from enucleated cells, thereby minimizing the potential risks associated with horizontal gene transfer [179]. The use of autologous exosomes in therapeutic drug delivery is an option for reducing undesired immunological side effects. Moreover, the stability and durability of exosomes in the systemic circulation are concerns. Circulating exogenous exosomes rapidly disappear within a half-life of 2–30 min and are taken up by macrophages associated with the mononuclear phagocytic system [180]. For the clinical application of therapeutic exosomes, their short residence times in circulation must be overcome. Therefore, exosome engineering is required to increase the lifetime of exosomes in circulation and reduce immune clearance. For example, the overexpression of CD47 on the surface of exosomes could increase their half-life threefold in circulation, allowing them to escape phagocytosis by circulating monocytes [181]. Surface molecules, such as PD-L1, CD31, and CD24, are also considered to have immune evasion capabilities, but they have not been applied in exosome-based drug delivery research; therefore, additional studies are needed.

Despite existing challenges and limitations, exosome utilization represents a promising approach in the field of drug delivery. This technology is still in its early stages, but holds great potential for the precise and targeted treatment of various diseases that have a significant impact on human health. In the future, continued research will drive the advancement of exosome-based drug delivery, paving the way for personalized medicine to treat diverse diseases.

## Data Availability

No new data were created.
